# SARS in Hospital Emergency Room

**DOI:** 10.3201/eid1005.030579

**Published:** 2004-05

**Authors:** Yee-Chun Chen, Li-Min Huang, Chang-Chuan Chan, Chan-Ping Su, Shan-Chwen Chang, Ying-Ying Chang, Mei-Ling Chen, Chien-Ching Hung, Wen-Jone Chen, Fang-Yue Lin, Yuan-Teh Lee, the SARS Research Group of National Taiwan University College of Medicine and National Taiwan University Hospital

**Affiliations:** * National Taiwan University Hospital, National Taiwan University College of Medicine, Taipei, Taiwan

**Keywords:** Severe acute respiratory syndrome, healthcare workers, environmental contamination, real-time reverse transcriptase–polymerase chain reaction

## Abstract

Thirty-one cases of severe acute respiratory syndrome (SARS) occurred after exposure in the emergency room at the National Taiwan University Hospital. The index patient was linked to an outbreak at a nearby municipal hospital. Three clusters were identified over a 3-week period. The first cluster (5 patients) and the second cluster (14 patients) occurred among patients, family members, and nursing aids. The third cluster (12 patients) occurred exclusively among healthcare workers. Six healthcare workers had close contact with SARS patients. Six others, with different working patterns, indicated that they did not have contact with a SARS patient. Environmental surveys found 9 of 119 samples of inanimate objects to be positive for SARS coronavirus RNA. These observations indicate that although transmission by direct contact with known SARS patients was responsible for most cases, environmental contamination with the SARS coronavirus may have lead to infection among healthcare workers without documented contact with known hospitalized SARS patients.

The coronavirus responsible for the severe acute respiratory syndrome (SARS-CoV) rapidly spread from Mainland China to 30 countries worldwide (*1*–*4*). From November 1, 2002, through July 31, 2003, a total of 8,098 probable cases were reported, including 346 from Taiwan (*2*). The disease is of great concern because of the high case-fatality rate, short incubation period, rapid spread along international air routes, and the large number of cases in previously healthy hospital staff (*1*,*2*,*5*–*7*). SARS appears to be spread most commonly by close person-to-person contact through exposure to infectious droplets and possibly by direct contact with infected body fluids (*1*,*5*–*7*). Emerging evidence indicates that SARS can be acquired from contaminated inanimate objects in the environment (*8*).

Taiwan is geographically close to China and Hong Kong and has a population of 23 million. An outbreak began on April 23, 2003, at a municipal hospital (hospital A) in Taipei. The index patient had unrecognized SARS. Multiple patients, visitors, and healthcare workers were exposed to this patient (*9*). After the outbreak at hospital A, patients sought care at the National Taiwan University Hospital, and patients with febrile illness screened in the emergency room (ER) increased substantially.

On May 8, 2003, we identified and reported to the local health department three SARS cases in patients whose only contact history was being treated at the National Taiwan University Hospital ER. Source and contact tracing failed to identify the index patient. In response to this outbreak, we admitted all ER patients in phases to a special unit where droplet and contact precautions were implemented, and on May 12, 2003, the operation of the ER was suspended.

On the same day, the infection control team was informed that three healthcare workers who worked in the ER had fever. They were immediately isolated, and initial interviews with the healthcare workers failed to identify a common source of infection. To better understand the mode of transmission, we conducted this epidemiologic study and environmental surveillance by using a highly sensitive and specific assay for SARS-CoV RNA. We describe how we traced the index patient to hospital A and the subsequent occurrence of three clusters of SARS after exposure to the National Taiwan University Hospital ER. We also provide evidence for indirect-contact transmission among some of the healthcare workers on the basis of the environmental studies.

## Materials and Methods

### Hospital Setting

The National Taiwan University Hospital is a 2,400-bed teaching hospital that provides both primary and tertiary care. National Taiwan University Hospital is located in downtown Taipei not far from hospital A. The ER is organized into several sections, including triage, examination, observation, critical care, and a clean area reserved for staff activities. A SARS screening unit was established on March 17, 2003, to interview and triage febrile patients with suspected cases of SARS. The patients were questioned about the presence of fever, myalgia, gastrointestinal or respiratory symptoms, whether they had close contact with a SARS patient, and recent travel. N-95 respirators were placed on patients suspected to have SARS early during the triage process, and they were immediately placed in private rooms (SARS area) to avoid contact with others in the ER. A daily record was maintained of all patients screened in the SARS screening unit. SARS cases were defined according to the World Health Organization criteria (*10*), modified to expand the definition of contact to include any healthcare setting with nosocomial transmission.

### Infection-Control Measures

Since March 14, 2003, infection-control measures required that all healthcare workers who had contact with patients with SARS use personal protective equipments, including gown, gloves, N-95 respirators, disposable cap, and shoe covers. Later, a face shield was included for healthcare workers with close contact to SARS patients.

Healthcare workers who had any contact with SARS patients or their environment were placed under surveillance for 14 days after the last exposure. Those who had **unprotected exposure,** or those who were protected but had high-risk exposures to SARS patients were excluded from new duty assignments and were restricted from direct patient care and contact with other healthcare workers. Any healthcare worker in whom fever developed was placed in specially designated isolation wards.

Infection-control measures in the non-SARS area were upgraded stepwise in response to possible healthcare-associated transmission and the increasing possibility of community spread of SARS. After the outbreak in hospital A, healthcare workers wore N-95 respirators for all patient care in the ER. In the ER, SARS areas were cleaned at least three times; non-SARS areas were cleaned once a day.

### Identifying Cases and Sources of Exposure

We obtained source and contact information for all patients identified at our hospital as having suspected or probable SARS. After the outbreak in hospital A, health insurance records were used to trace prior visits to other hospitals. In addition, we obtained information about social, hospital, and occupational contacts and other members of the household who had been exposed to suspected SARS patients within 10 days before the onset of their symptoms. All close contacts exposed to SARS patients during the period from 2 days before the onset of fever to the time of isolation were traced to identify the need for quarantine. Inpatients who had close contact with SARS patients were quarantined in private rooms, and contact and droplet precautions were implemented. On April 30, because of the occurrence of two closely spaced cases of SARS in the ER observation unit (a non-SARS area), we immediately identified a potential outbreak. Accordingly, we screened all inpatients that had been admitted through the ER and telephoned all the patients who stayed in the observation unit from April 23 through April 29.

Soon after the cluster of SARS was identified among healthcare workers on May 12, we devised a questionnaire to identify the source and the factors contributing to infection. Data about daily exposures were collected from April 30 through May 12, 2003. These data included contact with SARS patients; work areas; day, time, and characteristics of duty; exposure to high-risk aerosol-generating procedures; use of personal protective equipment; hand hygiene practices; and contact with other healthcare workers who did not use N-95 respirators.

### Environmental Survey

On May 15, surfaces of environment and equipment were sampled with moistened sterile cotton swabs. The swabs were spread immediately onto 3 mL of viral transport medium. Samples were collected from various objects in different areas of ER.

Air samples were taken with both high-volume and low-volume samplers in 10 locations in the ER. We used a high-volume air sampler (XMX Virtual Impactor, Dycor Technologies Ltd., Alberta, Canada) to draw air at calibrated sampling rates of 400 L per min for 5 min into a collector with 5 mL of phosphate-buffered saline (PBS). We also used a low-volume pump (Sidekick, SKC Inc., Eighty Four, PA) to draw air at a calibrated sampling rate of 2.0 L per min for 10 h onto a 37-mm diameter, 0.3-μm pore size polytetrafluoroethylene membrane filters. The collected samples were then frozen at –70°C before RNA extraction. Once environmental contamination was identified, cleaning was performed. Follow-up surveillance for contaminated objects was conducted on May 25.

### Viral Molecular Testing

Swab samples were suspended in 5 mL of PBS or 3 mL of viral transport medium. Total RNA from 140 μL of the sample was extracted by using a QIAamp Virus RNA Mini Kit (Qiagen, Hilden, Germany) and eluted in 60 μL of buffer. A volume of 5 μL of RNA solution was analyzed. RealArt HPA-Coronavirus LC RT-PCR Reagents (Roche, Penzberg, Germany) were used for one-step real-time reverse transcription–polymerase chain reaction (RT-PCR) in the Roche LightCycler Instrument (Roche, Mannheim, Germany). This ready-to-use system is designed for specific amplification of the 80-bp region of the SARS-CoV genome and for directly detecting the specific amplicon in fluorimeter channel F1 of the LightCycler Instrument. In addition, these reagents contain a second heterologous amplification system to identify possible PCR inhibition. Internal controls in each run of the experiment included two negative controls (one for RNA extraction and one for RT-PCR) and four quantification standards (1 x 10^1^ copies/μL, 1 x 10^2^ copies/μL, 1 x 10^3^ copies/μL, and 1 x 10^4^ copies/μL). We used the following formula to convert the values determined by using the standard curve into copies per milliliter of sample material: results (copies/mL) = result (copies/μL) x elution volume (μL)/sample volume (mL). Data were presented as number of copies per sample.

## Results

### Identification of Outbreaks in the ER

**Figure 1:**
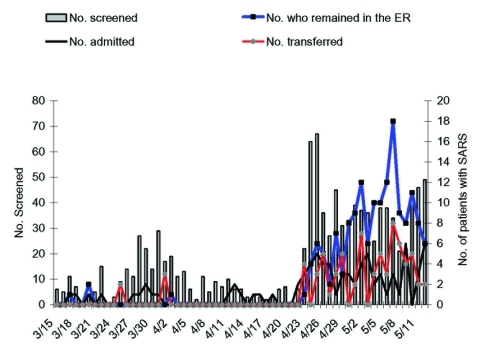
Time course during which patients with febrile illnesses were screened for severe acute respiratory syndrome (SARS) (vertical bars) and patients with SARS were detected at the emergency room of National Taiwan University Hospital, March 15–May 12, 2003. The numbers of patients with SARS who were admitted to this hospital is shown in black lines. The number of patients who temporarily stayed in the emergency room or were transferred to other hospitals is shown in red and blue lines, respectively.

From March 15 through April 22, a median of 6 patients per day (range 0–29) were screened at the ER for febrile illnesses (Figure 1). After the outbreak in hospital A, a median of 36 patients per day (range 21–67) were screened. Thus, the ER was used to screen a large portion of persons during this rapidly progressing epidemic. Of 754 patients screened at the ER from April 23 through May 12, a total of 63 patients were identified as SARS cases and were admitted to National Taiwan University Hospital, 68 SARS patients were transferred to another hospital, and 155 received care in a temporarily designated ER area because of shortages of isolation rooms and staff. On May 7, up to 18 SARS patients stayed in the ER overnight. Of 232 SARS patients admitted to the National Taiwan University Hospital from March 14 through June 19, 31 (13.4%) did not have a history of travel, exposure to SARS patients, or a hospital visit within 10 days before illness, and the only contact history was a stay at the National Taiwan University Hospital ER.

### Source and Contact Tracing

**Figure 2:**
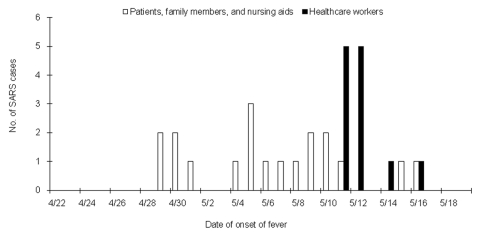
Epidemic curves showing three clusters of cases of severe acute respiratory syndrome (SARS) during the outbreak at the emergency room of the National Taiwan University Hospital. The first two clusters (open lines) consisted of patients, family members, and nursing aids. The third cluster (solid lines) consisted entirely of healthcare workers.

**Figure 3:**
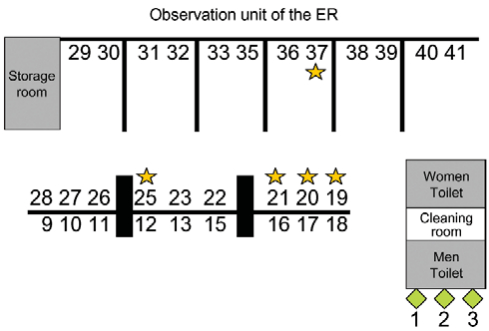
Allocation of bed numbers in the observation unit of patients involved in the first cluster (squares) and the second cluster (stars) of severe acute respiratory syndrome at the emergency room (ER) of National Taiwan University Hospital.

We identified three distinct clusters by plotting the dates of onset of fever for each case (Figure 2) and allocation of bed numbers in the observation unit of patients involved (Figure 3). The first cluster of five patients had disease onset from April 29 through May 1; the second cluster of 14 cases began on May 4, and the third cluster of 12 cases began on May 11, 2003. In the third cluster, all the cases were in healthcare workers. The first cluster affected patients located in three neighboring beds in the observation unit of the ER (Figure 3). The second cluster affected patients located in four nearby beds and a fifth bed that was >3 m away. The distance between beds was approximately 1 m. None of the cases occurred in beds 9–18, which are separated by a half wall.

**Figure 4:**
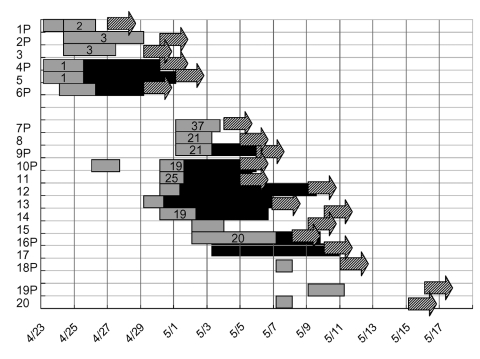
Contact history and temporal relationships among contacts according to the date of fever for 19 cases of severe acute respiratory syndrome (SARS) during the first two clusters of SARS at the emergency room of National Taiwan University Hospital. On April 27, fever and pneumonia developed in the index patient (patient 1) of the first cluster. The second cluster from an unknown source was identified on May 8. P, patients. Unlabeled numbers indicate family members or nursing aids. Location in the emergency room is shown in gray bars and in general wards by black bars. Numbers within the gray bar represent bed numbers in the observation unit of the ER. The dates of onset of fever are shown in arrows.

The index patient in the first cluster was an afebrile 73-year-old man who was admitted to the ER because of severe dyspnea (Figure 4, 1P). He was kept in the observation unit from April 23 through April 25, 2003. He was thought to have congestive heart failure and chronic obstructive lung disease and treated with aerosolized medication. He was admitted to the cardiology ward on April 25. A temperature of >38°C developed on April 27, and a chest radiograph taken on the same day indicated a new infiltrate. He was immediately transferred to a negative-pressure isolation room. He had not given this history, but after checking his health insurance card, we learned that he had visited hospital A on April 14 and April 15. He died on April 30. Sputum samples were positive for SARS-CoV RNA. On autopsy, he was found to have had an acute myocardial infarction. A small ground-glass density in the lung was compatible with viral pneumonitis.

The second patient was another 73-year-old man who had cough and fever for 2 weeks. He had been treated in the ER observation unit from April 24 through April 29. He returned to the ER on April 30 and was diagnosed with probable SARS. Because of the occurrence of two closely spaced cases of SARS in the ER observation unit (a non-SARS area), we immediately identified a potential outbreak. Contact tracing identified a cluster of cases in three patients and two nursing aids (Table 1, patient 2–6; Figure 4). All five cases were diagnosed as probable SARS, and results of three tests were positive for SARS-CoV RNA. The contacts were quarantined, and no tertiary cases emerged.

**Table 1:** Medical histories and conditions of the index patient and 19 patients affected in the clusters of severe acute respiratory syndrome related to the emergency room (ER) of National Taiwan University Hospital

Patient no.	Age/sex	Characteristics
Index patient		
1	73/M	Coronary artery disease, recent percutaneous occlusive balloon angioplasty and stenting, congestive heart failure, chronic obstructive lung disease, diabetes mellitus, chronic renal insufficiency. Had dyspnea without fever.
First cluster		
2	73/M	Infarction, hypertension, diabetes mellitus, old cerebral vascular accident, parkinsonism, hyperthyroidism. Cough for 2 weeks and fever for 1 week and was treated for aspiration pneumonia.
3	24/F	Nursing aid of patient 2.
4	62/F	Hepatitis C virus-related cirrhosis of liver, hypertension, diabetes mellitus. Had upper gastrointestinal bleeding.
5	64/F	Nursing aid of patient 4. Bronchial asthma.
6	65/F	Common bile duct stone and obstructive jaundice status post endoscopic retrograde cholangiopancreatography and endoscopic nasobiliary drainage, gallbladder stone status post cholecystectomy. Had fever and abdominal pain.
Second cluster		
7	88/M	Chronic obstructive pulmonary disease, hypertension, coronary artery disease status post percutaneous transluminal coronary angiography. Had lower intestinal bleeding secondary to ischemic colitis.
8	46/F	Family member of patient 9.
9	71/F	Acute pancreatitis, diarrhea.
10	65/F	Coronary artery disease status post percutaneous transluminal coronary angioplasty, major depression, diabetes mellitus, hypertension, end-stage renal disease under regular hemodialysis at a regional hospital. Persistent fever, diarrhea, leukocytosis and normal chest radiograph.
11	63/F	Took care of her son with acute pancreatitis in the ER.
12	38/M	Took care of his mother with end-stage renal disease undergoing hemodialysis. Stayed in the ER for 2 hours on April 29. Fever developed on May 7. Chest radograph was abnormal on May 11.
13	48/F	Nursing aid of a patient with pancreatitis close to acute patient 10 had frequent diarrhea. She helped take care of patient 10.
14	43/F	Nursing aid of patient 10
15	24/F	Nursing aid of a patient who visited the ER on May 2.
16	46/F	Pancreatic cancer with liver metastasis, perforated gastric ulcer status post primary closure and duodenostomy, gastrostomy and jejunostomy, poor control of diabetes mellitus and hypertension, glaucoma. Abdominal pain and watery diarrhea and was diagnosed as adhesion ileus and subcutaneous abscess caused by *Klebsiella pneumonia*.
17	43/F	Nursing aid. Contact to patient 9. Colon tubular adenoma status post polypectomy, chronic paranasal sinusitis status post functional endoscopic sinus surgery.
18	28/M	Tinea pedis, cellulitis.
19	69/M	Coronary artery disease, status post percutaneous transluminal coronary angioplasty. Abdominal discomfort and loss of appetite for several weeks. Cholangiocarcinoma, obstructive jaundice, biliary tract infection and upper gastrointestinal bleeding were diagnosed.
20	28/F	Nursing aid.

The second cluster began on May 8. A 46-year-old, otherwise-healthy woman (patient 8) was admitted with probable SARS. A week earlier she had taken care of her mother (patient 9) in the ER observation unit. She indicated that she did not have contact with other SARS patients, including those identified in the first cluster. Accordingly, we screened all patients who stayed in the observation unit from April 30 through May 8. This cluster affected six patients, three family members, and five nursing aids (Figures 2 and 4; Table 1, patients 7–20). Patient 17 was the only tertiary case.

The third cluster was noted on May 12, when the infection control team was informed that fever developed in three healthcare workers who had been isolated. The exact contact source could not be identified. Thus, we quarantined all the ER healthcare workers and suspended ER operations for 2 weeks. SARS related to the ER developed in 12 healthcare workers from May 11 through May 16 (Figure 2, solid lines). Six of the healthcare workers who became ill had close contact with SARS patients. However, patient contact and time of exposure were different. The healthcare workers were one desk clerk, two physicians, one radiology technician, and two nurses. All had followed infection-control precautions. Six other healthcare workers who became ill indicated that they did not have close contact with SARS patients. These workers were four nurses and two cleaners. These 12 healthcare workers differed from each other according to duty pattern, service time, work areas, and time of exposure to the unit (data not shown). Source and contact tracing failed to identify a common source. We therefore postulated that they might have acquired SARS through indirect contact.

### Environmental Survey

On May 15, we collected 119 environment samples, including 100 surface samples and 19 air samples (Table 2). Nine samples were positive for SARS-CoV RNA. These included the buttons of the drinking water fountains in the triage and the observation unit; a bedside chair in the observation unit; the outlet of the central air supply; a table top, bedding and bed edge in a SARS area; and a bookshelf and bedding in the clean area. None of 19 air samples tested positive for viral RNA. The highest viral load was obtained from a bedside chair in the observation unit (2,570 to 25,700 copies per sample).

**Table 2:** Results of environmental surveillance for severe acute respiratory syndrome (SARS) coronavirus RNA determined by real-time reverse transcriptase–polymerase chain reaction

Source of samples	No. of samples collected	No. (%) of positive samples	Source of positive result (copies of viral RNA per sample)
Surface of environment			
Triage	11	1 (9.1)	Button of drinking water fountains (257–2,570)
Examination area	10	0	
Observation unit	42	2 (4.8)	Button of drinking water fountains (257–2,570) Bedside chair (2,570–25,700)
Critical care area	3	0	
SARS area	10	4 (40.0)	Outlet of central air supply (257–2,570) Table top (257–2,570) Bedding (257–2,570) Bed edge (257–2,570)
Clean area	14	2 (14.3)	Book shelf (257–2,570) Bedding (257–2,570)
High-efficiency particulate air filter	10	0	
Air			
High-volume sampler	9	0	
Low-volume samples	10	0	
Total	119	9 (7.6)	

### Control Measures and Follow-up

Targeted cleaning of the ER environment was performed. Follow-up surveillance was conducted on May 25. Nine samples were collected from previously contaminated surfaces, 21 samples from other areas in the ER, and 15 samples from SARS wards. All 45 samples were negative for SARS-CoV RNA. All personnel who had contact with SARS patients or their environments were reeducated on infection-control measures. Particular attention was paid to hand hygiene and routine environmental cleaning. The workload for healthcare workers was reduced. All patient beds were placed at least 2 m apart. No further cases of SARS related to the ER occurred after May 17, 2003.

## Discussion

This report describes three clusters of SARS cases related to exposure to the ER at National Taiwan University Hospital during the epidemic in Taiwan. The index patient had been exposed to SARS at a nearby hospital. The patients symptoms were atypical for SARS, and he initially indicated that he had not been to hospital A. He had chronic cardiac and pulmonary disease and was afebrile. To date, healthcare-associated acquisition of SARS has been reported from eight hospitals in Taiwan (including National Taiwan University Hospital). All have been linked to the initial outbreak at hospital A (*9*). Unrecognized cases of SARS are probably the most important factor that led to intrahospital spread and cases among healthcare workers (*11*,*12*).

Most patients appear to have acquired their infections by close patient contact, presumably by droplet transmission. Six of the cases among the healthcare workers had no direct SARS patient contact. They may have acquired their infection from commonly used, contaminated objects. Finding SARS-CoV RNA in nine commonly used inanimate objects supports this notion. Although the signal only demonstrated SARS-CoV RNA and not viable virus, this finding may indicate that the virus can persist in the environment.

Environmental contamination was first demonstrated during a community outbreak in Hong Kong (*13*). The SARS virus may be stable in the environment at room temperature for 1 to 2 days (*8*). It can survive on plastic surfaces, stainless steel, glass slides, and paper files. The virus can survive even longer (up to 4 days) in stool from patients with diarrhea (*8*). In some series, diarrhea is a common complaint of SARS patients (*14*). One patient (patient 2) in the first cluster had intestinal bleeding, and 4 of 14 patients in the second cluster had diarrhea.

Overcrowding in the ER during an epidemic creates more opportunities for cross transmission and environmental contamination. In addition, overworked medical staff may not follow preventive procedures and take inadequate precautions (*15*,*16*). After the outbreak in hospital A, healthcare workers in the ER wore N-95 respirators for all patient care. Using protective equipment may account for the absence of cases among healthcare workers during the first and the second clusters of SARS in the ER. However, the third cluster included six healthcare workers who were not exposed to patients with SARS. Thus, masks do not prevent acquisition from environmental sources. Furthermore, the spread of SARS was most likely facilitated by lack of proper handwashing than by direct contact with patients or environments contaminated with viral nucleic acids. Therefore, intensive environmental cleaning should be instituted as soon as a case is identified, particularly for those with diarrhea. In addition, the importance of handwashing cannot be overemphasized.

This study has several limitations. Comprehensive serologic surveys were not conducted among all of the healthcare workers and patients during the outbreak. We may have missed persons with subclinical or mild infections who might have transmitted SARS by person-to-person contact. Viral cultures were not performed on samples taken from inanimate objects. SARS virus detected by RT-PCR may not have been viable.

Epidemiologic data suggest that transmission of SARS is mainly through close contact with droplets or secretions. But increasingly epidemiologic evidence, including this report, shows that the disease may also be transmitted indirectly through contact with hands or objects contaminated with secretions or excreta from patients with diarrhea. Clarifying the route of transmission will help prevent nosocomial transmission and allay fears that protection is inadequate.

## References

[1] World Health Organization Multicentre Collaborative Network for Severe Acute Respiratory Syndrome (SARS) Diagnosis. A multicentre collaboration to investigate the cause of severe acute respiratory syndrome. Lancet. 2003;361:1730–3. 10.1016/S0140-6736(03)13376-4 12767752 PMC7119328

[2] World Health Organization. Summary of probable SARS cases with onset of illness from 1 November 2002 to 31 July 2003. [Accessed Sept 26, 2003] Available at http://www.who.int/csr/sars/country/table2003_09_23/en/

[3] Ksiazek TG, Erdman D, Goldsmith CS, Zaki SR, Peret T, Emery S, A novel coronavirus associated with severe acute respiratory syndrome. N Engl J Med. 2003;348:1953–66. 10.1056/NEJMoa030781 12690092

[4] Drosten C, Günther S, Preiser W, van der Werf S, Brodt HR, Becker S, Identification of a novel coronavirus in patients with severe acute respiratory syndrome. N Engl J Med. 2003;348:1967–76. 10.1056/NEJMoa030747 12690091

[5] Tsang KW, Ho PL, Ooi GC, Yee WK, Wang T, Chan-Yeung M, A cluster of cases of severe acute respiratory syndrome in Hong Kong. N Engl J Med. 2003;348:1977–85. 10.1056/NEJMoa030666 12671062

[6] Poutanen SM, Low DE, Henry B, Finkelstein S, Rose D, Green K, Identification of severe acute respiratory syndrome in Canada. N Engl J Med. 2003;348:1995–2005. 10.1056/NEJMoa030634 12671061

[7] Lee N, Hui D, Wu A, Chan P, Cameron P, Joynt GM, A major outbreak of severe acute respiratory syndrome in Hong Kong. N Engl J Med. 2003;348:1986–94. 10.1056/NEJMoa030685 12682352

[8] World Health Organization. First data on stability and resistance of SARS coronavirus compiled by members of WHO laboratory network [Accessed May 4, 2003]. Available from: http://www.who.int/csr/sars/survival_2003_05_04/en/index.html

[9] Lee ML, Chen CJ, Su IJ, Chen KT, Yeh CC, King CC, Severe acute respiratory syndrome—Taiwan, 2003. MMWR Morb Mortal Wkly Rep. 2003;52:461–6. 12807078

[10] World Health Organization. Case definitions for surveillance of severe acute respiratory syndrome (SARS) (revised May 1, 2003) [Accessed May 4, 2003]. Available at http://www.who.int/csr/sars/casedefinition/en/

[11] Fisher DA, Lim TK, Lim YT, Singh KS, Tambyah PA. Atypical presentations of SARS. Lancet. 2003;361:1740. 10.1016/S0140-6736(03)13336-3 12767755

[12] Leo YS, Chen M, Heng BH, Lee CC, Paton N, Ang B, Severe acute respiratory syndrome—Singapore, 2003. MMWR Morb Mortal Wkly Rep. 2003;52:405–11. 12807088

[13] Tomlinson B, Cockram C. SARS: experience at Prince of Wales Hospital, Hong Kong. Lancet. 2003;361:1486–7. 10.1016/S0140-6736(03)13218-7 12737853 PMC7134636

[14] Peiris JSM, Chu CM, Cheng VCC, Chan KS, Hung IFN, Poon LLM, Clinical progression and viral load in a community outbreak of coronavirus-associated SARS pneumonia: a prospective study. Lancet. 2003;361:1767–72. 10.1016/S0140-6736(03)13412-5 12781535 PMC7112410

[15] Seto WH, Tsang D, Yung RWH, Ching TY, Ng TK, Ho M, Effectiveness of precautions against droplets and contact in prevention of nosocomial transmission of severe acute respiratory syndrome (SARS). Lancet. 2003;361:1519–20. 10.1016/S0140-6736(03)13168-6 12737864 PMC7112437

[16] Ofner M, Lem M, Sarwal S, Vearncombe M, Simor A. Cluster of severe acute respiratory syndrome cases among protected health-care workers—Toronto, Canada, April 2003. MMWR Morb Mortal Wkly Rep. 2003;52:433–6. 12807083

